# Breaking the Symmetry of a Metal–Insulator–Metal-Based Resonator for Sensing Applications

**DOI:** 10.1186/s11671-022-03684-6

**Published:** 2022-04-19

**Authors:** Chung-Ting Chou Chao, Yuan-Fong Chou Chau, Hai-Pang Chiang

**Affiliations:** 1 Department of Optoelectronics and Materials Technology, National Taiwan Ocean University, Keelung, 20224 Taiwan; 2 Centre for Advanced Material and Energy Sciences, Universiti Brunei Darussalam, Tungku Link, Gadong, Negara, BE1410 Brunei Darussalam

**Keywords:** Biosensor, Circular-shaped resonator, Metal–insulator–metal waveguide, Finite element method

## Abstract

This article designed a novel multi-mode plasmonic sensor based on a metal–insulator–metal waveguide side-coupled to a circular-shaped resonator containing an air path in the resonator. The electromagnet field distributions and transmittance spectra are investigated using finite element method-based simulations. Simulation results show that an air path in the resonator's core would impact the transmittance spectrum of SPPs. Besides, the air path is crucial in offering efficient coupling and generating multiple plasmon modes in the sensor system. The proposed structure has the advantage of multi-channel, and its sensitivity, figure of merit, and dipping strength can reach 2800 nm/RIU, 333.3 1/RIU, and 86.97%, respectively. The achieved plasmonic sensor can also apply for lab-on-chip in biochemical analysis for detecting the existence or nonappearance of diabetes through the human glucose concentration in urine.

## Introduction

Surface plasmon polaritons (SPPs) are the surface resonant excitations, including electromagnetic (EM) wave and collective electronic motions simultaneously, that the excitation happens at the interface of metal–dielectric boundary [1–9]. SPP waves have broad-ranging applications in optical devices and integrated optical circuits (IOCs) due to their advantage of overcoming diffraction limits and confining the light within the subwavelength regime [10–15]. As a result, different configurations of optical devices depending on SPP waveguides have been investigated and designed, such as absorbers [16, 17], filters [18, 19], amplifiers [20, 21], switches [22, 23], sensors [24–28]. Among them, SPP-based metal–insulator–metal (MIM) waveguides with strong light trapping, low ohmic loss, cost-effective fabrication, and long traveling path have attracted many research groups’ consideration [29, 30]. MIM-cavity waveguide-based structure can design the plasmonic refractive index (RI) sensor due to the ease of compatibility with IOCs, compact size, and susceptible feature to a small change of ambient medium [31, 32].

Near-Infrared (NIR) spectroscopy is a potential analytical method that can get information on most chemical specimens with the merit of low power energy, less influence by heat and fluorescence, and nondestructive and label-free [33, 34]. However, the drawback of mismatching between sample sizes and NIR wavelength leads to limit sensitivity and spatial resolution [35]. MIM-cavity waveguide can solve this mismatch because the light fields can restrict and enhance in the nanoscale resonator [36]. However, the research field in NIR is rarely discussed based on MIM-cavity waveguides before, and this topic requires further investigation.

Resonance cavities with diverse shapes undergo a pivotal role in offering a preferable light-matter interaction in the MIM-cavity waveguide system [37, 38]. Recently, many research groups proposed various MIM-cavity schemes for constructing the plasmonic sensors, e.g., rectangular-shaped [39, 40], circular-shaped [41–43], elliptical-shaped [44, 45], crossed ring-shaped [46, 47], T-shaped [48, 49] cavities, and many other frameworks. The circular-shaped cavity is the most popular one due to the smooth surface, ease of fabrication, and small filling factor in a unit area [50, 51]. This paper reports a multimode plasmonic sensor based on a MIM bus waveguide side coupled to a circular-shaped resonator, including a rotational air path in the inner core working in the NIR wavelength range. We investigated and compared three configurations of side-coupled resonators, i.e., case 1 (one circular-shaped cavity), case 2 (one circular-shaped ring resonator), and case 3 (case 2 with an air path in the resonator’s inner core), respectively. The finite element method (FEM) has been employed to analyze transmittance resonance modes and EM field distributions. In the case 3 structure, we use a rotational air path set in the resonator’s core instead of a circular one to break the resonator’s symmetry, which would impact the transmittance spectrum of SPPs. Modifying the geometry in the resonator's core can ameliorate the sensing performance. It is found that the air path can play a crucial role in providing efficient coupling between the bus waveguide and the resonator, breaking the structural symmetry, and offering an additional optical way in the proposed plasmonic system. Unlike the previously reported works, e.g., a horizontal air path and a vertical air stub [52] set in the resonator’s inner core, the proposed case 3 structure possesses the merit of rotational air path, which can offer an additional degree of freedom (i.e., the rotational angle of the air path, *θ*) to facilitate the coupling effect between the bus waveguide and resonator. In addition, the proposed plasmonic sensor can also be applied for testing glucose concentration in human urine because it is easily accessible and is no bleeding. In practical situations, patients with diabetes must bleed blood for glucose testing, enduring pain, and causing uncomfortable. The novelty of this work is that we have measured the glucose concentration level in the urine specimens to detect diabetes in patients with 0.001 RI variation. To the best of our knowledge, we studied this issue using the SPPs MIM-cavity-based plasmonic sensor for the first time.

## Methods and Fundamental

**Fig. 1:**
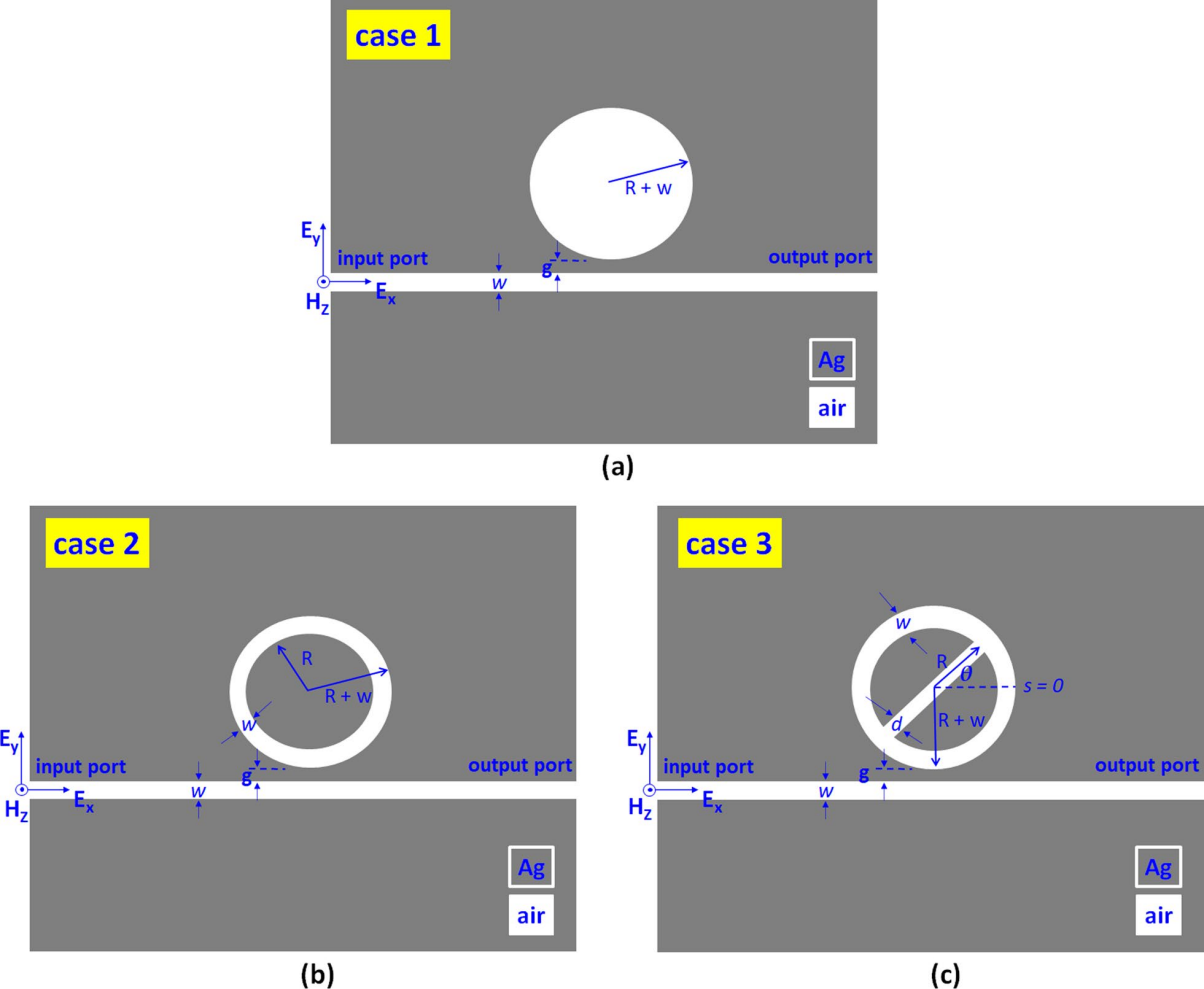
Top view of the investigated plasmonic sensors, consisting of a MIM bus waveguide coupled with one circular ring-shaped cavity. **a** case 1: without an inner core, **b** case 2: with an inner core, and **c** case 3: with an air path in the inner core, respectively

Figure 1a–c illustrates the top view of three sensor cases, i.e., case 1: a MIM bus waveguide (width *w*) side-coupled to a circular air cavity (radius *R *+ *w*), case 2: case 1 with an inner core (radius *R*), and case 3: case 2 with an air path (width *d*) in the inner core, respectively. We indicated the structural parameters in Fig. 1a–c. They are the gap distance between the bus waveguide and the circular-shaped cavity (*g*), the displacement of the air path along the y-axis (*s*), and the angle of the air path (*θ*, an angle between the x-axis and the center of air path), respectively. Note that s shifts the centers of the air path along the + y- axis or − y- axis. In Fig. 1, the gray- and white-colored regions represent the silver (Ag) and air. A TM-polarized EM wave coupled with the fundamental SPP mode [53–56] into the bus waveguide's input port, and the transmission power can reach the output port. The Drude model can describe Ag's frequency-dependent permittivity (*ε*_*m*_) [57, 58].

1 $$\varepsilon_{m} \left( \omega \right) = \varepsilon_{\infty } - \frac{{\omega_{p}^{2} }}{{\omega^{2} + i\omega \gamma }}$$

where *ε*_∞_ = 3.7 is the infinite dielectric constant, *ω* stands for the frequency, *ω*_p_ = 9.10 eV is bulk plasma frequency, and *γ* = 18 meV is the electron collision frequency, respectively.

A 2D physical model replaces the 3D physical model because the structure height in the z-axis is much larger than the skin depth of SPPs in the x- and y- axes. COMSOL Multiphysics with the mesh size of ultrafine mesh grid size with the number of degrees of freedom of 68,815 to maintain the convergence of the results. Perfectly matched layers use to absorb the outgoing waves without reflection around the outer boundaries of the simulation domain. When the EM field impinges into the input port, it will reflect and transmit some energy, and part of it will couple into the ring resonator. The amount of reflected, transmitted, and coupled energy depend on the degree of coherent coupling and interference between the bus waveguide and the resonator. The circular-shaped ring resonator can serve as a Fabry–Pérot cavity, and the resonance will occur when the SPPs are side-coupled into the ring resonator and satisfy the resonance condition in the resonator. We can call the transmission modes as original modes of the ring resonator [59]. The SPPs can be excited when the incident EM wave approaches the intrinsic resonance wavelength (*λ*_res_). If Δ*φ* = 2π*m* (*m* is an integer), the *λ*_res_ can be expressed by temporal coupled-mode theory [60, 61].

2 $$\lambda_{{{\text{res}}}} = \frac{{2L_{{{\text{eff}}}} {\text{Re}} (n_{{{\text{eff}}}} )}}{{m - \frac{\varphi }{2\pi }}} \left( {m = 1,2,3 \ldots } \right)$$

Here *m* denotes the order of the standing wave resonance, *L*_eff_ represents the effective length of the resonator, φ stands for the phase shift, and Re(*n*_eff_) is the real part of the effective RI. *n*_eff_ can describe as:

3 $${\text{Re}} (n_{{{\text{eff}}}} ) = \left( {\varepsilon_{{{\text{silver}}}} + \left( {\frac{k}{{k_{0} }}} \right)^{2} } \right)^{1/2}$$

where *k* = 2*π*/*λ* is the wave vector, *k*_0_ is the wave vector in the free space, and *ε*_silver_ is the silver’s permittivity.

**Fig. 2:**
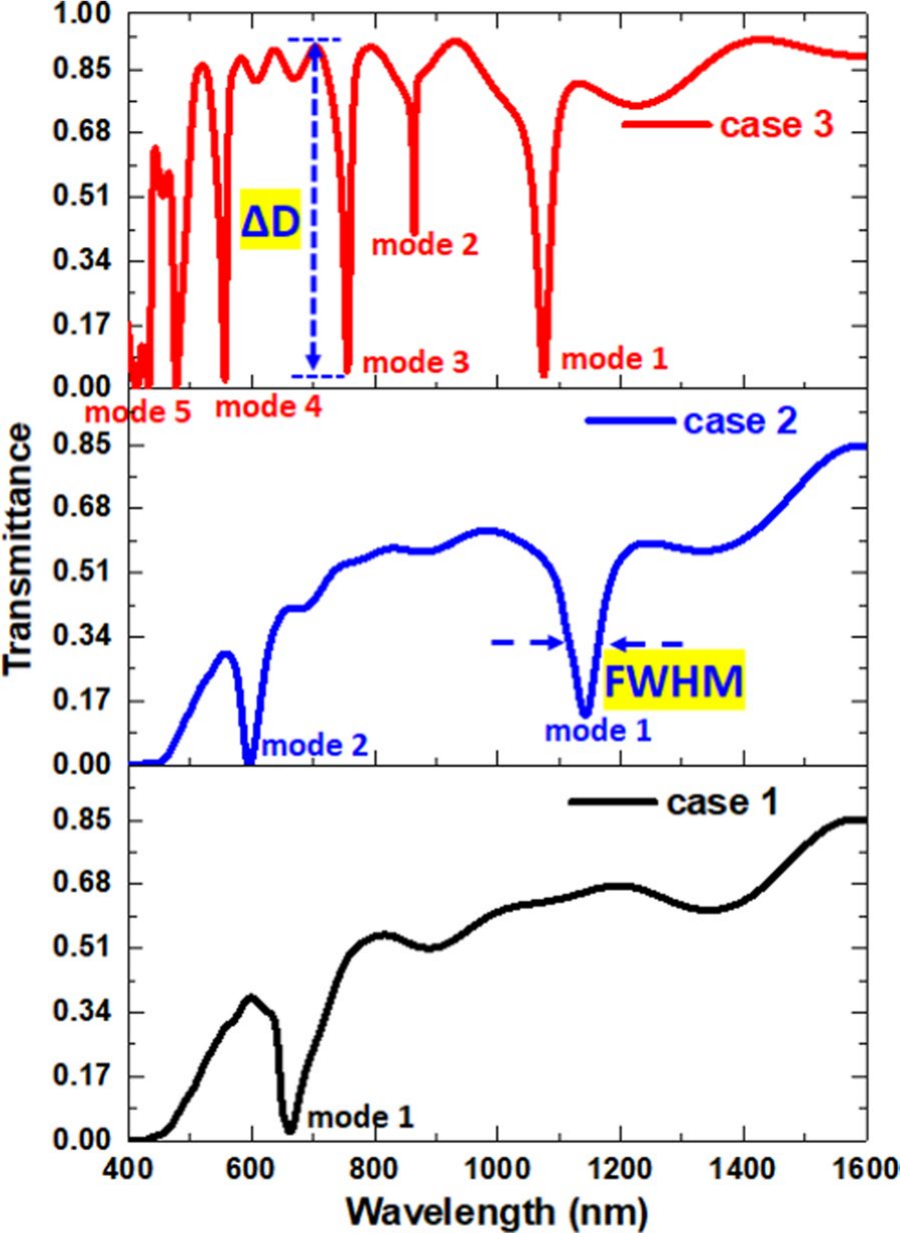
Comparison of the transmittance spectrum of the SPPs mode for cases 1–3 structures

The input/output ports are located at the left/right ends of the designed device (see Fig. 1) to monitor the input/output powers. The transmittance (T) can obtain by *T* = *P*_out_ (output power)/*P*_in_ (input power), where the *P*_out_ and *P*_in_ can calculate as integral values of energy flux density. The FWHM is full width at half-maximum, and the quality factor (QF) stands for the quality coefficient and can express as Eq. (4). The sensitivity (S) can calculate from Eq. (5). Besides, the figure of merit (FOM) can define by Eq. (6).

4 $${\text{QF}} = \lambda_{{{\text{res}}}} {\text{/FWHM}}$$

5 $$S = \Delta \lambda_{{{\text{res}}}} {/}\Delta n\left( {{\text{nm/RIU}},\;{\text{nanometer}}\;{\text{per}}\;{\text{RI}}} \right)$$

6 $${\text{FOM}} = S{\text{/FWHM}}$$

where Δ*λ*_res_ is the shift of the *λ*_res_, and Δ*n* is the change of RI. Besides, we define the dipping strength (ΔD) in Eq. (7), i.e., the difference between the transmittance peak and dip [62]; see the inset of Fig. 2.

7 $$\Delta D = (T_{{{\text{peak}}}} {-}T_{{{\text{dip}}}} ) \times 100\%$$

## Results and Discussion

Figure 2 compares the transmittance spectrum for cases 1–3 structures. To guarantee that only the TM mode can travel in the investigated structure, we keep the bus waveguide width as *w* = 50 nm throughout this paper. The default structural parameters, *R*, *g*, *s, θ*, and *d*, are 100 nm, 10 nm, 0 nm, 0°, and 50 nm, respectively. The resonator size of the proposed structure is compact and much smaller than many reported designs (e.g., [63, 64]). As seen, a distinct difference of the transmittance spectrum concerning the different resonance modes can elucidate this discrepancy after an air path appears in the proposed structure. The transmittance dips will redshift with the increase of the RI of filling dielectric [54]. The transmittance of the slit alone (i.e., only the bus waveguide in the plasmonic sensor system) exceeds 80% with the oscillating pattern in the wavelength range of 400–1600 nm [65], indicating that the incident light can transmit from the input port to the output port. As seen in Fig. 1, only one resonance mode occurred in case 1, which is associated with the original mode between the bus waveguide and resonator. In case 2, we found two transmittance dips corresponding to mode 1 and mode 2 in the wavelength range from 400 to 1600 nm, respectively. The two SPP modes attribute to the surface plasmon resonance (SPR) and cavity plasmon resonance (CPR) from the coupling effect between the bus waveguide and circular ring resonator [66–68]. When an air path exists in the resonator’s core, case 3 can produce more SPP modes since the enhanced SPR and CPR, resulting in five SPP modes corresponding to mode 1 to mode 5, respectively. It will break the symmetry of the circular-shaped ring resonator with an air path instead of a split circular core, which could alter the propagation path of SPPs in the resonator. Note that the overall transmittance at off-resonance in case 3 is much higher than cases 1 and 2. This result can be attributed to the significant destructive interference (i.e., less light-matter interaction) between the bus waveguide and resonator at off-resonance, demonstrating the lower ohmic loss in the case 3 structure [69]. We compared the *λ*_res_, FWHM, ΔD, S, FOM, and QF of cases 1–3 at corresponding resonance modes in Table 1. For testing the sensitivity, the RI value (*n*) is from 1.00 to 1.05 with an interval of 0.01. The interference of SPR and CPR causes the multiple SPP modes among bus waveguide and circular-shaped ring resonator. According to Fig. 2 and Table 1, we found that the air path acts as a critical role in offering a more significant number of plasmon modes, enhancing the coupling effect, breaking the structural symmetry, and creating an additional optical path. We can conclude that the resonance dip in case 3 has a more profound dip strength (∆D), a narrower FWHM, and a higher QF than the other cases. These remarkable merits could help to improve the RI-sensing performance. This noticeable characteristic of the case 3 structure gives way to the possible applications in nanophotonics devices.

**Table 1:** Comparison of *λ*_res_, FWHM, S, FOM, ΔD, and QF of cases 1–3 structures at resonance modes

	Mode 1	Mode 2	Mode 3	Mode 4	Mode 5
**Case 1**					
*λ*_res_ (nm)	663				
FWHM (nm)	45.00				
ΔD (%)	15.61				
S (nm/RIU)	600				
FOM (1/RIU)	13.33				
QF	14.73				
**Case 2**					
*λ*_res_ (nm)	1143	596			
FWHM (nm)	40.00	20.00			
S (nm/RIU)	1100	500			
FOM (1/RIU)	27.5	25.00			
ΔD (%)	32.68	29.52			
QF	28.58	29.80			
**Case 3**					
*λ*_res_ (nm)	1075	865	756	556	447
FWHM (nm)	20.00	3.00	10.00	10.00	10.00
S (nm/RIU)	1100	800	700	500	400
FOM (1/RIU)	55.00	266.67	70.00	50.00	40.00
ΔD (%)	80.90	53.11	85.81	85.30	86.18
QF	54.30	29.10	76.30	56.10	48.10

For testing the sensitivity, the RI value (*n*) is from 1.00 to 1.05 with an interval of 0.01

**Fig. 3:**
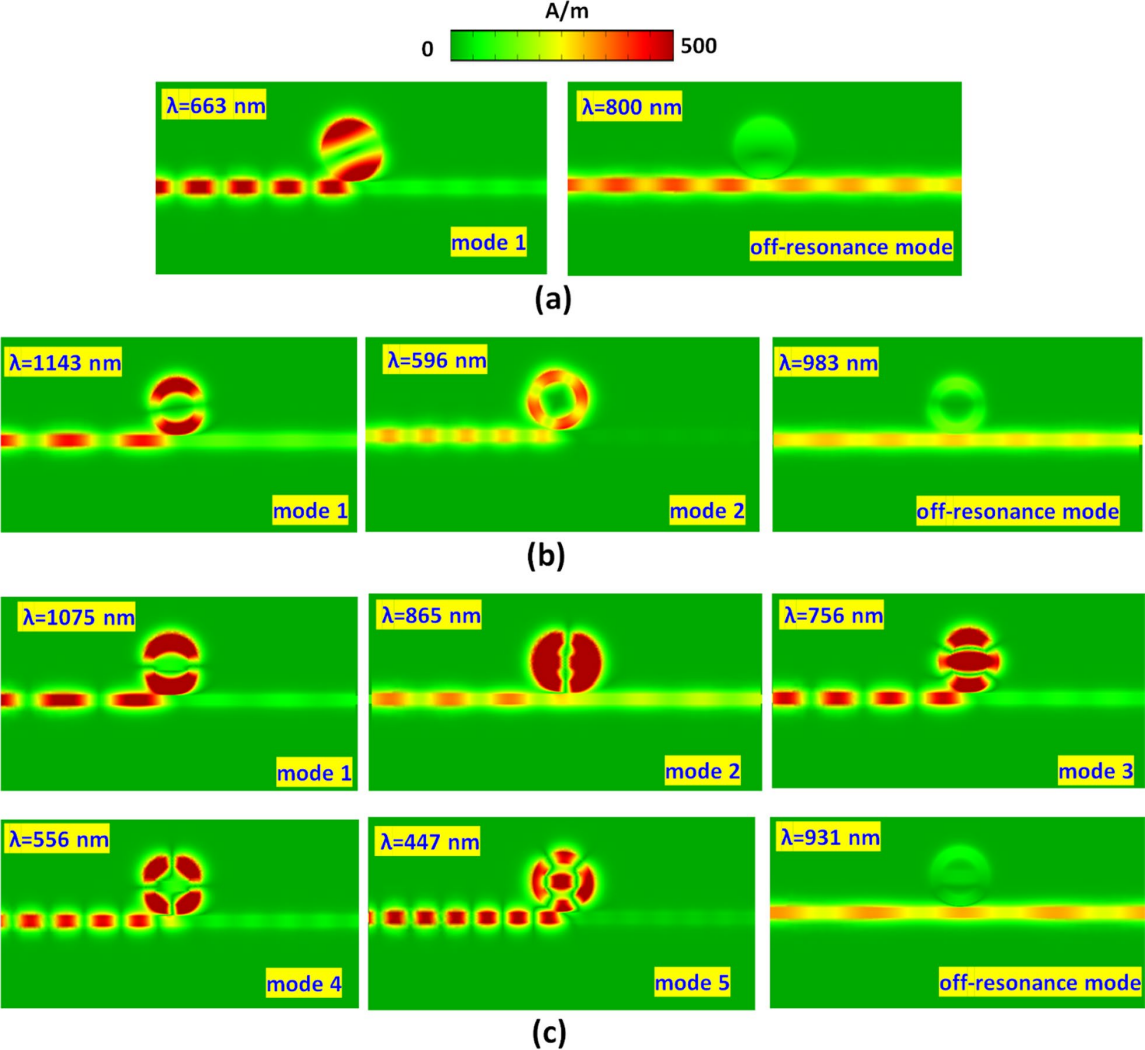
Truncate views of magnetic field intensity (|H|) at the corresponding wavelengths of resonance modes and off-resonance modes in **a** case 1, **b** case 2, and **c** case 3, respectively

To go into the physical nature, Fig. 3a, b illustrates the steady state of the magnetic field intensity (|H|) at the corresponding wavelengths of resonance modes and off-resonance modes in case 1 and case 2, respectively. As seen, the standing wave occurs in the circular-shaped ring resonator, and most input EM wave traps in the circular-shaped ring resonator at *λ*_res_. The incident wavelength highly influences the |H| patterns of SPP modes due to the different phase and wave number [44, 70]. The light spot number of |H| fields in circular-shape ring resonator are two for mode 1 in case 1, two, and four for modes 1–2 in case 2, and two, two, three, four, and five for modes 1–5 in case 3, respectively. The different spot number attributes to the variant phase significantly perturb the bus waveguide and circular-shaped ring resonator. For example, the shorter incident wavelength can experience more wavelengths in a fixed optical path, resulting in more light spot numbers. Therefore, we can find five light spots in mode 5 of the case 3 structure. The SPPs wave can confine in resonator well because of the constructive interference between the bus waveguide and the circular-shaped ring resonator, revealing remarkable CPR. The |H| field enhancement of the SPP modes exhibit an excellent light-matter coupling in the circular-shaped ring resonator. In Fig. 3a, b, the |H| fields are hardly trapped in the circular-shaped resonators at off-resonance mode due to the destructive interference between the bus waveguide and circular-shaped ring resonator, showing the higher transmittance values as observed in Fig. 2.

**Fig. 4:**
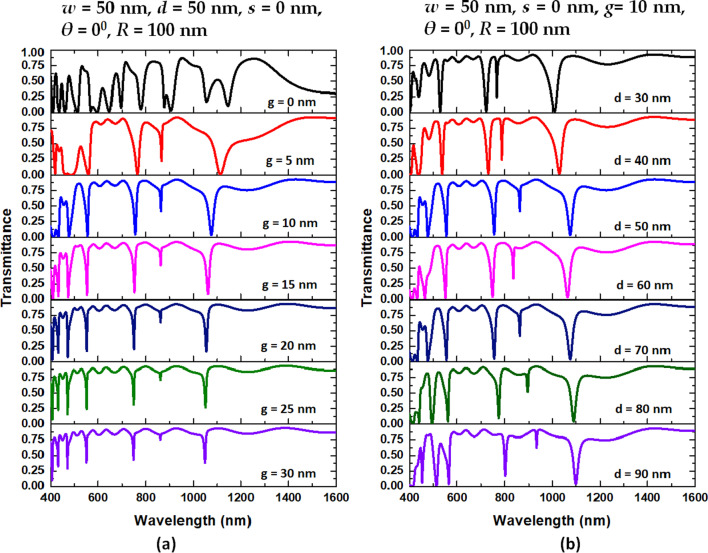
Transmittance spectra of the case 3 structure with **a** variation of *g* and **b** variation of *d*, respectively

Next, we inspect the four structural parameters, i.e., *g*, *d*, *θ*, *s,* and *R*, that have a relatively significant influence on the optical properties of case 3 while keeping the other value of parameters intact. The default parameters of *w, g*, *d*, *θ*, *s*, and *R* are 50 nm, 10 nm, 50 nm, 0 nm, 0°, and 100 nm. First, we inspect the influence of the variation of *g* and *d* of the case 3 structure on the transmittance spectrum, as shown in Fig. 4a, b, respectively. As clearly observed in Fig. 4a, b, the transmittance dips of mode 1 blueshifts with the increasing *g* (from 1145 to 1048 nm) and *d* (from 1099 to 1009 nm), while the transmittance dips of other modes change slightly. The raising *g* diminishes the coupling effect between the bus waveguides and the circular-shaped resonator. As seen, the transmittance profiles display a fierce oscillation due to a more significant coupling effect when *g* = 0 nm. Furthermore, the ∆D and FWHM significantly reduce with the increase of *g* for mode 2 since a declining coupling effect as the extending value of *g*. Thus, the *d*’s value can alter the cavity’s resonance condition and offer an optical path connected to both sides of the inner core. This feature hints that the balance of power flow strength of the discrete and the continuum state’s SPPs mode is changed by varying *d*, the resonance conditions in the air path are affected. According to Fig. 4a, b, the available ranges of *g* and *d* based on the *λ*_res_, transmittance curve shape, ∆D and FWHM are 5 < *g* < 25 nm and 30 < *d* < 90 nm, respectively, which reveals the reliability and robustness in the fabrication of proposed case 3 structure.

**Fig. 5:**
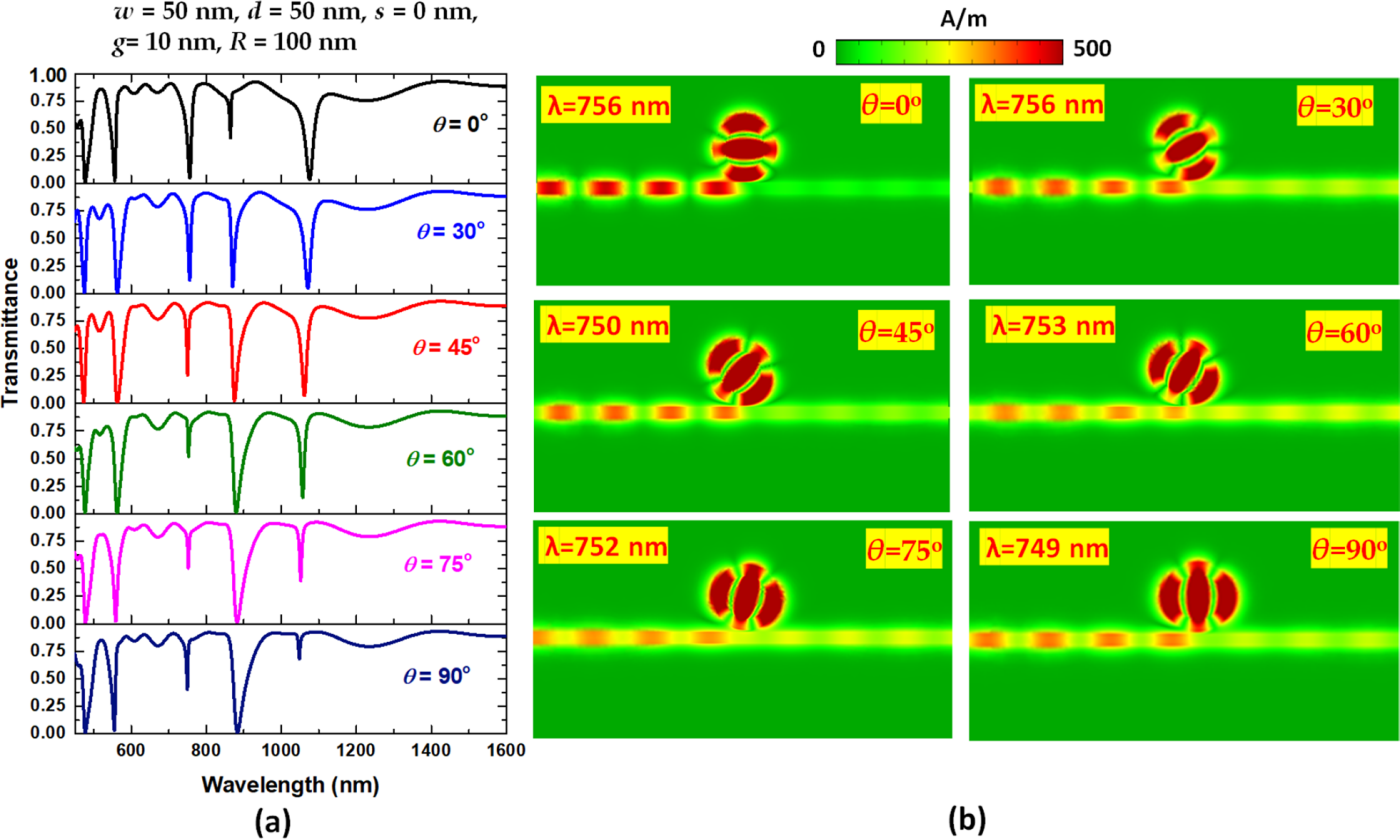
Transmittance spectra of the case 3 structure with **a** variation of *θ* and **b** selected magnetic field (|H|) intensity at the corresponding *λ*_res_ of mode 3

The coupling angle of EM wave between bus waveguide and the circular-shaped resonator can mediate the coupling effect and significantly influence the transmittance spectrum’s profile. Figure 5a, b depicts the transmittance spectrum of varying *θ* and the selected magnetic field (|H|) intensities at the corresponding *λ*_res_ of the case 3 structure. The transmittance spectra have different curve shapes to the variation of *θ* due to their different physical nature. We found five modes with variant ∆D in the 400–1600 nm wavelength range when *θ* varies from 0° to 90°. In Fig. 5a, the transmittance dips occur at *λ*_res_ when the air path has a rotational angle of *θ*. Compared with the symmetric structure (*θ* = 0°), the sensing performance of the asymmetric case 3 structure is greatly improved when *θ* increases from 0° to 30°. For the asymmetric structure (θ > 0°) because parts of EM wave locate at the magnetic nodes of the standing waves in the resonator, there is a transmittance dip [71]. As seen in Fig. 5a, the ∆D will rise with the increase of *θ* in modes 2 and 3, while the ∆D will reduce with the growth of *θ* in mode 1. The workable range of *θ* is 0° to 90°, and its optimal value is *θ* = 30° based on ∆D and the transmittance curve shape. Notably, the case of *θ* = 30° shows five dipper transmittance dips with ∆D in the range of 74.74–86.97% since this angle undergoes the preferential coupling angle between bus waveguide and circular-shaped ring resonator. It indicates that the proposed structure behaves as better light-matter coupling between the bus waveguide and resonator when *θ* = 30°. The air path with a rotational angle of *θ* = 30° provides the strong confinement of SPPs and constructive interference in the circular-shaped resonator. This finding attributes to the resonator's symmetry breaking, leading to the rotational air path's instinctive SPR and CPR modes. Compared to the case of *θ* = 90°, only two transmittance dips can obtain due to the vertical coupling angle between the bus waveguide and resonator. We summarized the *λ*_res_, FWHM, transmittance peaks (*T*_max._), transmittance dip (*T*_min_.), ∆D and QF of case 3 structure at *θ* = 30° in Table 2. Modifying the geometry in the resonator’s core can improve the sensing performance. One can conclude that the rotational angle of the air path in the resonator plays an essential role in breaking the structure symmetry and dominating the coupling efficiency between the bus waveguide and circular-shaped resonator.

**Table 2:** Comparison of *λ*_res_, FWHM, transmittance peaks (*T*_max._), and transmittance dip (*T*_min_.), ∆D and QF of case 3 structure at *θ* = 30°

	Mode 1	Mode 2	Mode 3	Mode 4	Mode 5
*λ*_res_ (nm)	1071	871	756	563	475
FWHM (nm)	20.00	10.00	8.00	12.00	10.00
*T*_max_	91.87%	91.88%	90.74%	80.71%	75.59%
*T*_min_	4.9%	6.5%	11.91%	0.80%	0.85%
ΔD (%)	86.97	85.38	78.83	79.91	74.74
QF	53.55	87.10	94.5	46.92	47.50

In Fig. 5b, the different incident wavelength influences the |H| field distributions at corresponding *λ*_res_ with different phases. The selected |H| fields at the corresponding *λ*_res_ of mode 3 in circular-shaped ring resonator show three petals of light spots, and the outline of air path match with the corresponding angle of *θ*. Besides, the dipolar effect could excite along both sides of the air path, i.e., resulting in positive–negative charge pairs. This phenomenon can dominate the field enhancement in the circular-shaped ring resonator. The air path in the inner circular-shaped core permits the highly trapped SPP modes and offers effective coupling efficiency and constructive interference in the resonator. Therefore, the apparent transmittance dips can observe in Fig. 5a.

**Fig. 6:**
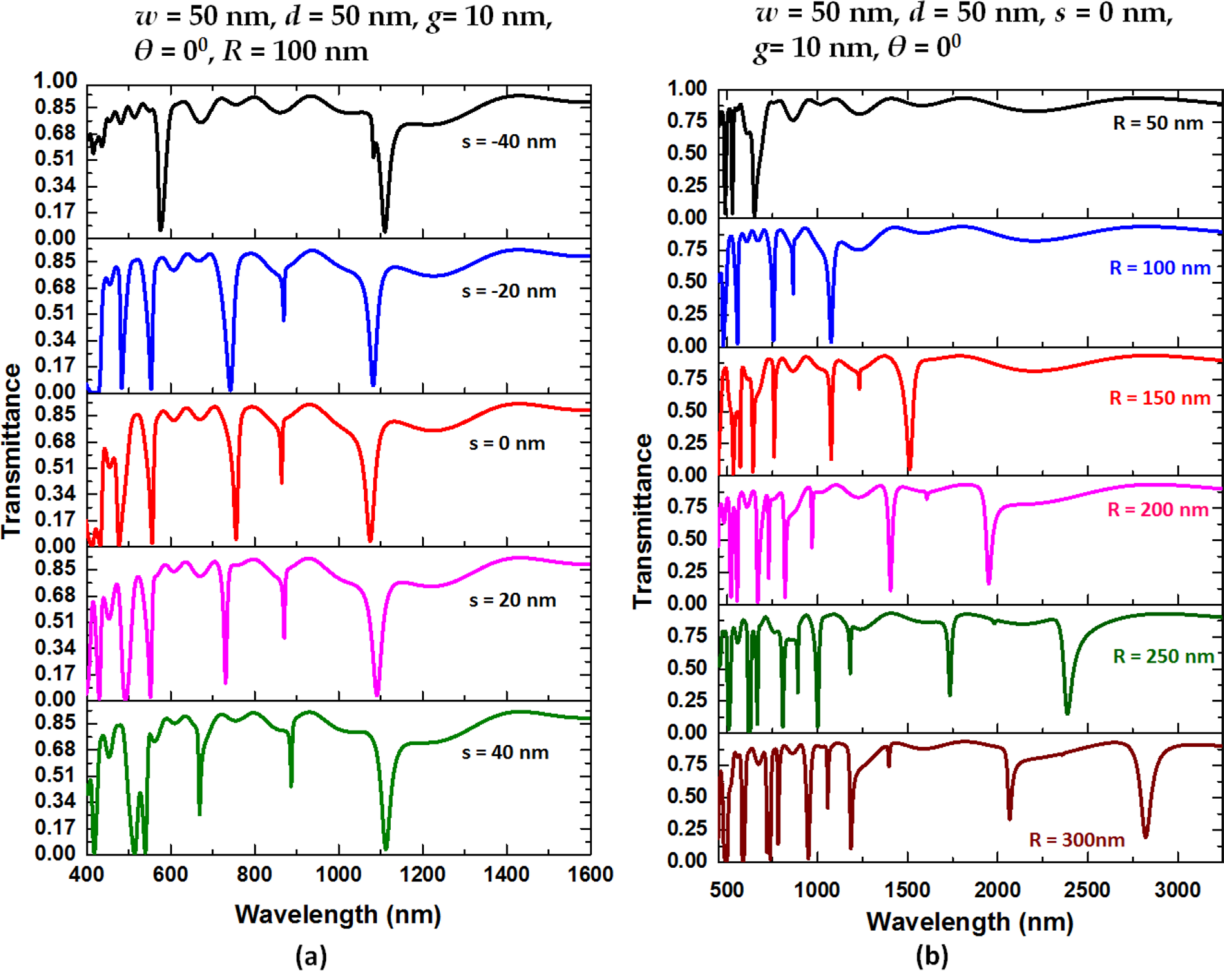
Transmission spectra versus **a** *s* variation and **b** *R* variation of the case 3 structure

Successively, we inspect the transmission spectra of case 3 structure by varying *s* and *R*, respectively. As shown in Fig. 6a, the suitable range of s is − 40 to 40 nm. The number of resonance modes is two for *s* =  − 40 nm, five for *s* =  − 20 and 0 nm, and six for *s* = 20 and 40 nm, indicating that a larger *s* with a larger distance between air path and bus waveguide will excite more resonance modes due to the enhanced CPR effect in the circular-shaped ring resonator. It is evident from Fig. 6b that the larger *R* can attain a greater mode number and offer flexibility in tuning transmittance’s curve profile. Compared to other structural parameters, we found that the transmittance dip exhibits a remarkable redshift as the increase of *R*. This is because of the rise of effective length (*L*_eff_) of the resonator, which is in good agreement with Eq. (2). We notice that the shift of *λ*_res_ by varying *R* is more sensitive than *θ*, *s*, *d*, and *g*. Hence, we can choose the specific transmittance dip to the characteristic wavelengths by varying *R*. The mode number is three, five, seven, seven, nine, and nine for *R* = 50, 100, 150, 200, 250, and 300 nm in the wavelength range of 450–3000 nm, accordingly. Table 3 displays the *λ*_res_, S, and FOM of the case 3 structure when *R* is varied from 50 to 300 nm with an increment of 50 nm in the 450–3000 nm wavelength range, respectively. The RI of surrounding media, *n*, is 1.00–1.04 with an interval of 0.01. The calculated S can reach 2800, 2100, 1300, 1100, 1100, and 800 nm/RIU, while the FOM can get 30.0, 105.0, 216.7, 110.0, and 160.0 for modes 1–5 when *R* = 300 nm. Besides, these values are superior to the published articles (e.g., [72, 73],). Based on Fig. 6b, the workable values of *R* are in the range of 50 nm < *R* < 300 nm. It will confront to prepare the proposed sensor when *R*’s value is too small (e.g., *R* < 50 nm), yet if *R*'s value is too big (e.g., *R* > 300 nm), the device’s size, ∆D, FWHM, and ohmic loss [74] will get large [75], which is then useless. Based on Fig. 6b, we can conclude that the size of *R* will significantly contribute to the sensitivity performance to the proposed case 3 structure and enhance the CPR effect in the coupled resonator. It should note that the resonance mode’s intensity during RI sensing (dipping strength, i.e., Δ*D* = *T*_peak_ − *T*_dip_) directly influences the sensing accuracy as it is easier to inspect the sensing signal with a strong resonance [62]. The more ohmic losses raised by a longer optical path since the confinement loss is inevitable in the plasmonic MIM waveguide. It is evident in Fig. 6b that the dipping strengths exhibit a little difference (e.g., ΔD varies from 87.5% to 77.5% for mode 1) when *R* varies from 50 to 300 nm. The coupling EM wave could well confine in the resonator, showing a bonding resonance mode [76]. This finding demonstrates that the proposed case 3 structure possesses the advantage of low ohmic loss [77]. Thus, one can flexibly tune the desired working wavelength by varying the *R*’s value in the range of 50 nm < *R* < 300 nm.

**Table 3:** The *λ*_res_, S, and FOM corresponding to the resonance modes of the case 3 structure when *R* is varied from 50 to 300 nm with an increment of 50 nm in the wavelength range of 450–3000 nm, respectively

Mode		1	2	3	4	5	6	7	8	9
*R* = 50 nm	*λ*_res_ (nm)	661	528	487						
	S(nm/RIU)	400	400	400						
	FOM(1/RIU)	13.3	66.7	50.0						
*R* = 100 nm	*λ*_res_ (nm)	1075	865	756	556	447				
	S(nm/RIU)	1100	800	700	500	400				
	FOM(1/RIU)	55.0	266.7	70.0	50.0	40.0				
*R* = 150 nm	*λ*_res_ (nm)	1512	1232	1076	759	642	572	533		
	S(nm/RIU)	1500	1300	1100	700	600	600	500		
	FOM(1/RIU)	50.0	260.0	110.0	87.5	75.0	150.0	71.4		
*R* = 200 nm	*λ*_res_ (nm)	1951	1405	970	820	729	554	520		
	S(nm/RIU)	1900	1400	900	800	700	500	500		
	FOM(1/RIU)	63.3	56.0	225.0	100.0	62.5	62.5	71.4		
*R* = 250 nm	*λ*_res_ (nm)	2387	1735	1182	1001	891	808	666	617	507
	S(nm/RIU)	2400	1700	1200	1000	1000	700	700	600	400
	FOM(1/RIU)	48.0	113.3	240.0	83.3	333.3	175.0	233.3	40.0	40.0
*R* = 300 nm	*λ*_res_ (nm)	2822	2026	1397	1186	782	737	719	588	495
	S(nm/RIU)	2800	2100	1300	1100	800	700	700	500	500
	FOM(1/RIU)	30.0	105.0	216.7	110.0	160.0	140.0	140	33.3	25.0

Diabetes is a lasting metabolic disturbance disease arising from blood glucose (blood sugar) levels, which seriously harms the nervous system, kidneys, eyes, and heart [78]. One can demonstrate that human urine samples will change with the glucose concentration (GC) levels [79]. In typical situations, the human body has glucose ranging from 0 to 15 mg per deciliter (mg/dl) [80]. However, GC levels in human urine will rise to the average range of 165–180 mg/dl due to glycosuria. In Ref. [80], Sani et al. numerically investigated the change of GC level with RI using nanocavity of photonic crystal waveguide [81]. Moreover, Mostufa and coauthors analyzed the different urine GC level samples using graphene-coated SPR-based biosensor [79]. Here, we will examine the sensing performance using the MIM-cavity waveguide-based structure (i.e., case 3) for the first time to the best of our knowledge.

**Fig. 7:**
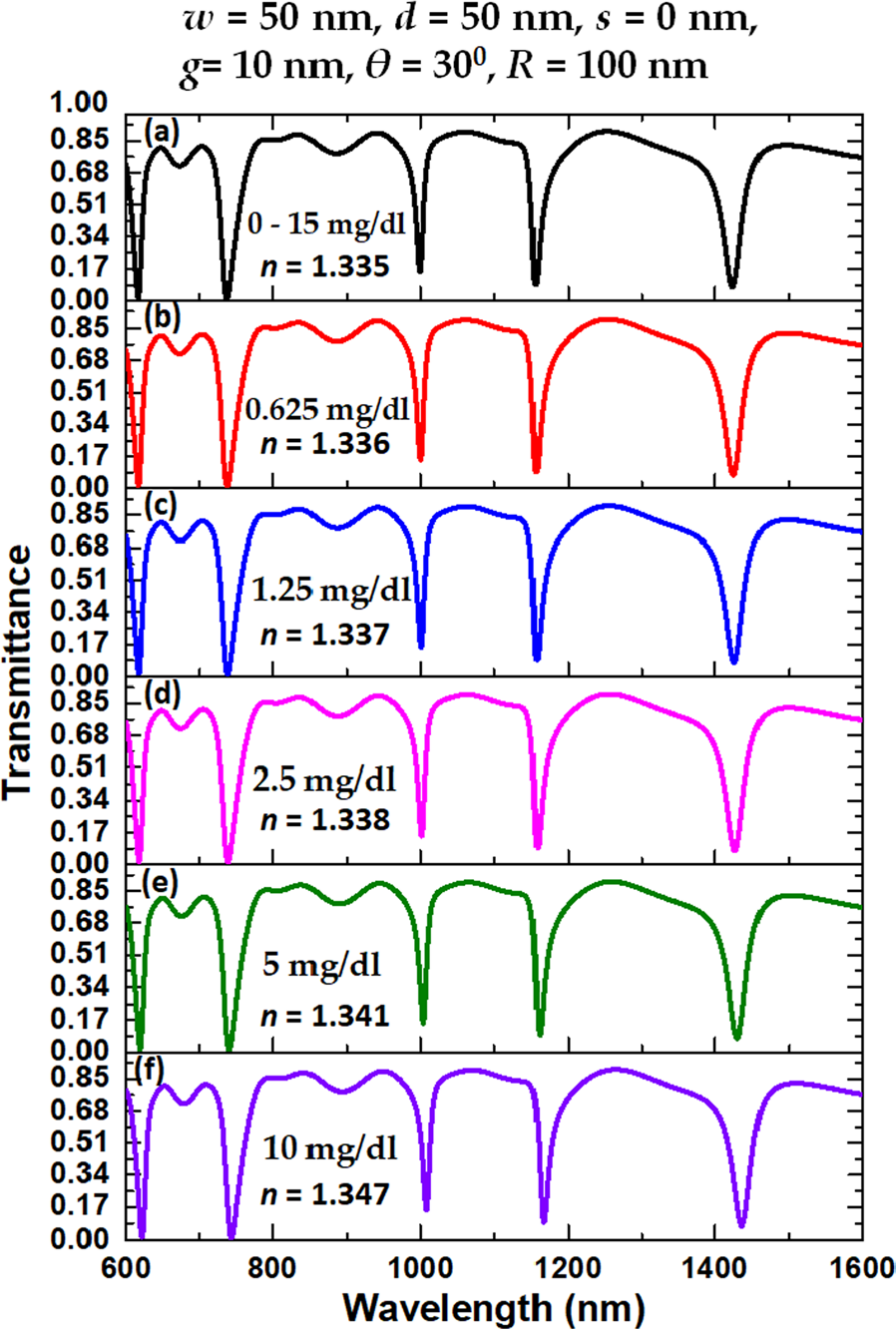
Transmittance spectra of case 3 structure at different urine glucose level samples, i.e., **a** 0–15 mg/dl (*n* = 1.335), **b** 0.625 mg/dl (*n* = 1.336), **c** 1.25 mg/dl (*n* = 1.337), **d** 2.5 mg/dl (*n* = 1.338), **e** 5 mg/dl (*n* = 1.341) and **f** 10 mg/dl (*n* = 1.342), respectively

**Fig. 8:**
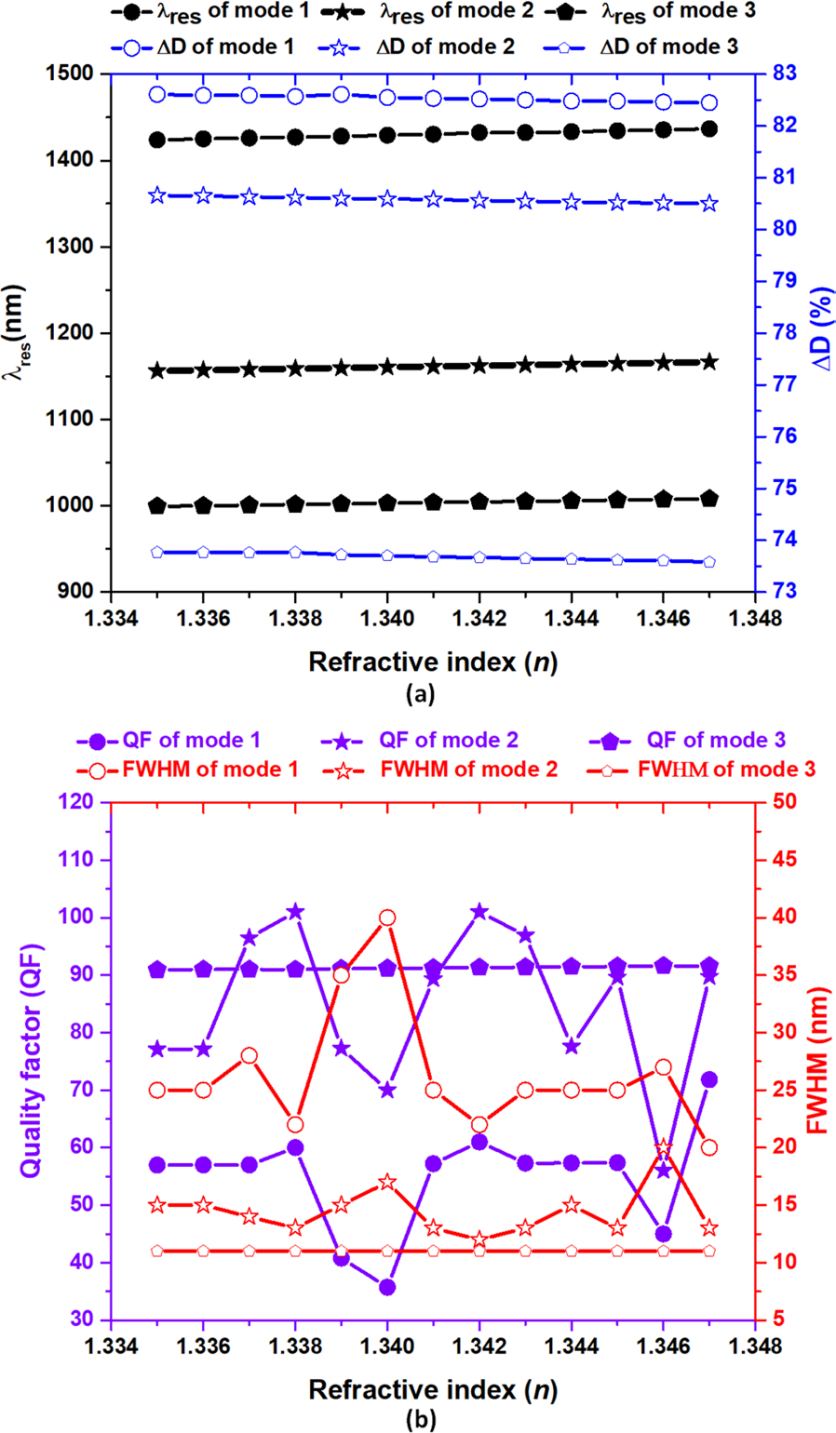
**a** *λ*_res_ and ∆D and **b** QF and FWHM versus the RI value (*n*) from 1.335 to 1.342 with the interval of 0.001 of case 3 structure for mode 1 to mode 3, respectively

Near-infrared (NIR) ranging in 0.75 ~ 3.0 μm is a low power intensity light that the irradiation effects are a response to the light but not to the heat [33]. Thus, it is suitable to detect the presence or absence of diabetes through the human GC in urine with specific *λ*_res_ ranging in NIR. This section will detect the GC levels in human urine using the proposed case 3 structure. Before that, we should select a suitable range of operation wavelengths. Infrared spectroscopy is a potential analytical method that can obtain information on the chemical composition of most specimens. As shown in Fig. 5a, the wavelengths corresponding to the case 3 structure at *θ* = 30° have three distinct resonance modes (i.e., modes 1–3) in this range. Thus, we adopted case 3 as the candidate based on excellent sensing performance and compact size (see Table 2). The sensor function of the case 3 structure utilizes the resonance cavity filled with specimens, and the same method can be applied to all cases of RI sensing. Depending on the RI of the human GC, each sample offers a specific resonance wavelength. As described in Ref. [82], diabetes has a very high RI. Filling the human urine specimens inside the circular-shaped resonator of the case 3 structure will lead to a RI shift (∆*n*) due to a GC level increment in urine samples. The plasmonic sensor would detect that by shifting the *λ*_res_, one can determine the main resonance modes in the case 3 structure by the circular-shaped resonator of a specific resonant wavelength concerning the GC in the human urine. Figure 7a–f depicts the transmittance spectrum of case 3 structure at different urine glucose level samples, i.e., 0–15 mg/dl (*n* = 1.335), 0.625 mg/dl (*n* = 1.336), 1.25 mg/dl (*n* = 1.337), 2.5 mg/dl (*n* = 1.338), 5 mg/dl (*n* = 1.341), and 10 mg/dl (*n* = 1.342), respectively. As seen, each case has five transmittance dips, and it can separate the shift of resonance wavelength (*λ*_res_), resulting in the normal person partitioning into the patient. The recently introduced detectors have a solution of detecting a wavelength shift as small as 0.1 nm. For the variation of GC in (0–15, 0.625) mg/dl (Fig. 7a, b), the corresponding *λ*_res_ shift from 1423.9–1425.0 nm for mode 1, 1156.3–1157.2 nm for mode 2, and 999.4–1000.1 nm for mode 3, to the ∆*n* of 0.001. The recorded (S, FOM) reach (1100 nm/RIU, 36.67 RIU^−1^), (900 nm/RIU, 180.00 RIU^−1^), and (700 nm/RIU, 70.00 RIU^−1^) corresponding to mode 1 to mode 3, accordingly. In the same manner, for two levels of GC in (1.25, 2.5) mg/dl in human urine (Fig. 7c, d), the corresponding *λ*_res_ change from 1426.0–1427.1 nm for mode 1, 1158.0–1158.9 nm for mode 2, and 1000.8–1001.6 nm for mode 3, to the ∆*n* of 0.001. The (S, FOM) achieve (1100 nm/RIU, 36.67 RIU^−1^), (900 nm/RIU, 180.00 RIU^−1^), and (800 nm/RIU, 80.00 RIU^−1^) corresponding to mode 1 to mode 3, accordingly. Similarly, for the case of GC in (5, 10) mg/dl in human urine (Fig. 7e, f), the corresponding *λ*_res_ vary from 1430.2–1436.7 nm for mode 1, 1161.4–1166.6 nm for mode 2 and 1003.8–1008.1 nm for mode 3, to the ∆*n* of 0.006. The (S, FOM) reach (1183 nm/RIU, 36.10 RIU^−1^), (866 nm/RIU, 173.20 RIU^−1^), and (716 nm/RIU, 71.60 RIU^−1^) corresponding to mode 1 to mode 3, accordingly. Therefore, we can detect the human urine samples by observing the *λ*_res_ shift in the transmittance spectrum using the proposed case 3 structure. Table 4 summarizes the *λ*_res_ (nm), S (nm/RIU), and FOM (1/RIU) of case 3 structure in different GC levels of patient urine samples corresponding to mode 1 to mode 3, respectively. Figure 8 also depicts *λ*_res_ and ∆D (Fig. 8a), and QF and FWHM (Fig. 8b) versus the RI value from 1.335 to 1.342 with the interval of 0.001 of case 3 structure corresponding mode 1 to mode 3. Besides, the sensor's ∆D is associated with the transmittance intensity (%) difference, which is closely related to the detecting accuracy and resolution. In Fig. 8a, as the RI increases, the *λ*_res_ increases linearly, and the recorded values of ∆D are in the range of 82.45–82.61% for mode 1, 80.50–80.60% for mode 2, and 73.58–73.77% for mode 3, showing the excellent values in modes 1–3. In Fig. 8b, we calculate the effects of RI changes on QF and FWHM. As seen, if the RI = 1.338 and 1.342, the QF reaches its highest value, and the two lowest values are found at RI = 1.340 and 1.346 for mode 1 and mode 2, while QF values are around 90.00 for mode 3, respectively. Therefore, we can conclude that the sensor structure for detecting GC levels in urine samples of the diabetic patient with a RI of 1.338 and 1.342 is highest and is minimized at a RI of 1.340 and 1.346, respectively. Besides, the FWHM values are in the range of 20–40 nm for mode 1, 12–15 nm for mode 2, and around 5–10 nm for mode 3; these values guarantee the accurate measurement of the GC level in urine samples due to the low FWHM with the best average bandwidth wavelength. Besides, the proposed case 3 structure can also serve as a temperature sensor to detect the thermal medium. A change in the temperature leads to a variation in the RI of the sensing medium [83, 84]. As a temperature sensor, a liquid, e.g., ethanol, with high RI temperature coefficient (3.94 × 10^4^) is filled into the resonator and bus waveguide region, which is then sealed [85, 86].

**Table 4:** *λ*_res_ (nm), S (nm/RIU), and FOM (1/RIU) of case 3 structure in different GC of patient urine samples corresponding to mode 1 to mode 3

			Mode 1			Mode 2			Mode 3		
Glucose	RI	∆*n*	*λ*_res_	S	FOM	*λ*_res_	S	FOM	*λ*_res_	S	FOM
0–15 mg/dl	1.335	Ref	1423.9	Ref	Ref	1156.3	Ref	Ref	999.4	Ref	Ref
0.625 mg/dl	1.336	0.001	1425.0	1100	36.67	1157.2	900	180.0	1000.1	700	63.64
1.25 mg/dl	1.337	Ref	1426.0	Ref	Ref	1158	Ref	Ref	1000.8	Ref	Ref
2.5 mg/dl	1.338	0.001	1427.1	1100	36.67	1158.9	900	180.0	1001.6	800	72.73
5 mg/dl	1.341	Ref	1430.2	Ref	Ref	1161.4	Ref	Ref	1003.8	Ref	Ref
10 mg/dl	1.347	0.006	1436.7	1083	36.10	1166.6	866	173.2	1008.1	716	65.09

Comparison of the sensitivity and figure of merit between this work and selected published works is given in Table 5. Based on Table 5 and the simulation results mentioned above, the proposed case 3 structure's obtained sensitivity is remarkably higher than those of similar MIM designs reported in the literature.

**Table 5:** Comparison of the sensitivity and FOM between this work and previous similar works

Reference/year	Structure	Max. sensitivity (nm/RIU)	Max. FOM (1/RIU)
[87]/2017	Two circular cavities	840	100
[88]/2018	Side-coupled stub-hexagon resonators	550	178.00
[89]/2019	Rectangular ring resonator	1367	22.30
[26]/2020	Resonator with taped defects	1295	159.60
[90]/2021	Clockwork spring-shaped resonator	1210	191.16
This work	Resonator with a rotational air path	2800	333.30

## Conclusion

This study proposed a plasmonic sensor based on a side-coupled circular-shaped ring resonator in a MIM-cavity waveguide system for RI and biomedical sensor applications. Three cases of resonators are investigated and compared, i.e., case 1 (one circular-shaped cavity), case 2 (one circular-shaped ring resonator), and case 3 (case 2 with an air path in the resonator’s core), respectively. We analyzed transmittance resonance modes and EM field distributions in detail using FEM-based simulations. An air path set in the resonator's core instead of a circular core can break the resonator’s symmetry, impacting the transmittance spectrum of SPPs. It is found that the rotational angle of the air path in the resonator’s core plays a pivotal role in breaking the structure symmetry and dominating the coupling efficiency between bus waveguide and circular-shaped ring resonator. Modifying the resonator’s core geometry can enhance the sensing performance and keep the structure size unchanged. When *R* = 300 nm, the sensitivity, figure of merit, and dipping strength can reach 2800 nm/RIU, 333.3 1/RIU, and 86.97%, respectively. The proposed case 3 with *R* = 100 nm can detect a different glucose concentration level from a healthy person by human urine specimens for each 0.001 RI change. The sensitivity can simultaneously operate in multiple modes and reach above 700 nm/RIU in modes 1–3. The minimum FWHM is ~ 5.0 nm, and the maximum FOM is 80.0 1/RIU. Besides, the dipping strength shows the excellence values ranging in 74.74%–86.97% in modes 1–5, while the recorded Q factors are in the range of 46.92–87.10 in modes 1–5. The proposed sensor is a promising candidate for nanophotonics and biochemistry since its excellent sensing performance with multiple modes and broad operation wavelengths.

## Data Availability

Not applicable.

## Abbreviations

SPPs: Surface plasmon polaritons
EM: Electromagnetic
IOCs: Integrated optical circuits
MIM: Metal–insulator–metal
RI: Refractive index
NIR: Near-infrared
FEM: Finite element method
FWHM: Full width at half-maximum
QF: Quality factor
S: Sensitivity
FOM: Figure of merit
ΔD: Dipping strength
SPR: Surface plasmon resonance
CPR: Cavity plasmon resonance
|H|: Magnetic field intensity
GC: Glucose concentration
